# Textronic UHF RFID Transponder

**DOI:** 10.3390/s21041093

**Published:** 2021-02-05

**Authors:** Piotr Jankowski-Mihułowicz, Mariusz Węglarski, Mateusz Chamera, Patryk Pyt

**Affiliations:** 1 Department of Electronic and Telecommunications Systems, Rzeszów University of Technology, Wincentego Pola 2, 35-959 Rzeszów, Poland; p.pyt@prz.edu.pl; 2 Talkin’ Things, Al. Wilanowska 317, 02-665 Warsaw, Poland; mateusz.chamera@talkinthings.com

**Keywords:** RFID textronic transponder, UHF textronic tag, Internet of Textile Things (IoTT), Internet of Things (IoT), textronics, RFIDtex, sewn UHF antenna, impedance matching, inductive coupling system

## Abstract

In order to respond the growing interest towards radio frequency identification textile transponders, the authors propose a new approach to design radio frequency identification (RFID) devices by introducing the RFIDtex concept. The coupling system of inductive loops is implemented in the textronic structure with the RFID interface in order to split the transponder into two independently manufactured components. Then both modules can be easily integrated into the RFIDtex tag. The presented simulation and measurement results prove the concept of manufacturing a relatively small antenna in the form of a meandered dipole sewn in with a single thread, and further, that can be connected to the RFID chip through the coupling system without galvanic junctions. The achieved parameters clearly indicate that the tag can correctly communicate with the read/write device as well as the coupling between its both parts works properly, and the impedance matching is possible in this case. The possibility of confectioning products with electronic identification tags at the textile factory site and improved resistance to the impact of environmental conditions are the main advantage of the proposed approach to the RFID devices designing. The RFIDtex transponder idea proposed by the authors was restricted in the patent no PL 231291 B1.

## 1. Introduction

### 1.1. Purpose of the Work

The idea of Internet of Things (IoT) is mainly disseminated through the use of wireless systems such as radio frequency identification (RFID). Although the RFID technology is becoming more and more popular in everyday life, it is still not sufficiently implemented in management and logistics processes of the textile industry. The major barrier of introducing automatic identification into the successive stages of the product life (i.e., production, logistic, storage, shipping, trade, service, usage, maintenance, utilization, etc.) consists in costs of its implementation. When looking at today’s market of RFID devices, designers have at their disposal incompatible concepts and systems dedicated to apply to a single stage of the mentioned product life. Therefore, it is usually not economically profitable to implement an RFID system just only, for example, to improve production processes. However, if there was a uniform construction that could meet expectations of all entities interested in managing a given product, the implementation costs could be spread among all users. It is the main reason why the authors directed their interest towards radio frequency identification textile transponders (RFIDtex transponders and RFIDtex tag). The attractiveness of the conception is based on endless opportunities of integrating the RFIDtex tags with consumer goods including mainly casual, formal, and protective clothes, uniforms, linen, and other everyday items [1]. The benefits drown from using the RFIDtex idea consist in possibilities of using the same tags, permanently integrated with the objects, to build a comprehensive identification system covering the entire life cycle of textile products. Of course, new transponder constructions need to be enhanced because they have to survive chemical and physical hazards that are specific for the successive stages as well as the requirement of improved esthetic appearance can emerge. It increases the price of the single RFIDtex tag, but it also significantly improves the logistics processes beginning from a production stage, through quality control (including warranty service), wholesale and retail distribution, personalized advertisement for customers, automatic washing (even with automatic adjustment of the washing machine programs to the recognized type of laundry) as well as determination of degree of wear, and finally utilization.

The idea of the RFIDtex transponders (restricted in the patent) is presented in the dedicated Section 5. Generally, the RFIDtex structures and their operational functions fit into the concept of intelligent textiles (smart textiles) [2], especially textiles joined with electronics (e-textiles) [3]. In this way the conception of Internet of Textile Things (IoTT) can be developed.

The major prerequisite when designing RFIDtex devices is to elaborate textile antennas in a way that is invisible to users or that fulfill high aesthetic requirements. This forces to change the idea of traditional RFID systems, in which a tag is attached to an object in an additional logistic process, towards the concept of a transponder integrated straight into the textile product on the production lines [4,5]. Moreover, since identification, monitoring and sensing applications based on the wearable wireless nodes are increasingly used in the health and welfare centers, hotel and catering sectors, trade, and service of textiles as well as laundry industry [6,7], the need to create new structures of transponders arises. The possibility of using the RFIDtex allows designers to meet requirements of these applications and on the other hand forces the progress in manufacturing low-cost antennas integrated into fabrics by means used in the textile industry [8]. This objective can be achieved thanks to significant advancements that have been made in the textronics as well as in the radio frequency identification (RFID) technique over the last decade, especially in context of wireless body area networks (WBAN) [9,10,11,12,13,14,15].

Antennas can be fabricated using newly developed conductive materials (e.g., yarns, threads, or paints) or other modern textile processing and production methods, such as sewing, embroidering, coating, weaving, knitting, spinning, laminating, printing, or chemical treatment [16,17]. Sewing conductive thread is a simple method with a high potential in fabrication of textronics goods due to its compatibility with non-electronic textile processing on various fabric materials. This method allows designers to fully control and map shapes of antenna conductive structures. Cost savings can be achieved by sewing closed shapes without filling them as well as it can reduce the time spent on transponder manufacturing. For this reason, it is important to investigate the impact of sewn antennas (i.e., its pattern geometry mapping, RFID chip attachment method, and dielectric and geometric parameters of used materials) on operation of the RFIDtex transponders [18].

The possibility of impedance matching between an RFID chip and a textile antenna along with efficiency of antenna sewn with a single thread was also studied in the paper. The impact of the sewn pattern on the wireless performance of passive ultra-high frequency (UHF) RFID tags was examined. Unlike the typical transponders in which the chip is permanently attached (trough soldering, gluing, etc.) to the pads of antenna circuit [19], it was necessary to pay special attention to the innovative and unusual way of assembling both components in the proposed RFIDtex devices.

The textile antenna for the passive UHF RFIDtex transponder, sewn with conductive threads is characterized in Section 1.2 and a short look at the state of the art in the area under consideration in Section 1.3. The synthesis process of antenna dedicated to a commercially available type of RFID chip is discussed in Section 2. The particular attention is paid to the method of matching the antenna and the chip impedances (Section 2.1) as well as to designing the antenna with the coupling system (Section 2.2). The antenna model was carried out in the EMCoS Antenna VLab software tool (EMCoS, Tbilisi, Georgia) and the adequate assumptions are described in Section 2.3. The design process of samples is presented in Section 3.1 and measurement results performed at the preparatory stage are described in Section 3.2. In Section 3.3, the experimental laboratory stand and its equipment are revealed. The background and obtained results of investigations on RFIDtex transponder parameters are presented in detail in Section 3.4 and Section 3.5. The work is summarized in Section 4. In the last Section 5, the reader finds information about the patent resulting from the work reported in this manuscript.

### 1.2. Textile UHF RFID Transponder

**Figure 1:**
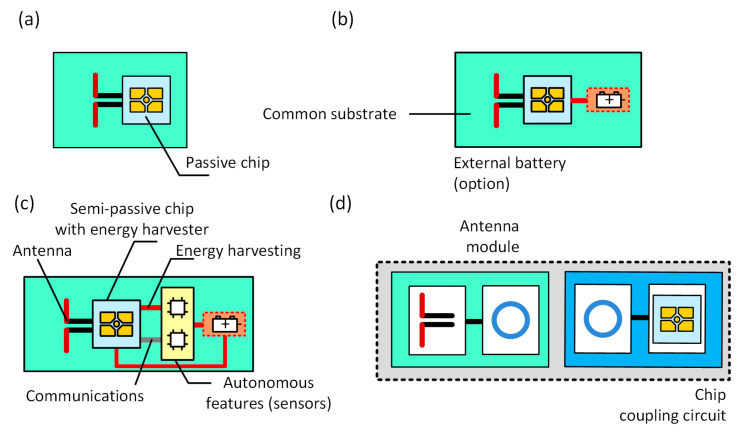
Block diagrams of UHF transponders: (**a**) Passive transponder; (**b**) Semi-passive transponder with external battery; (**c**) Semi-passive transponder with autonomous features and energy harvesting; (**d**) An innovative idea of transponder with separated chip and antenna.

The UHF RFID systems typically operate in the frequency range from 860 MHz to 960 MHz, as well as with selected frequency of 2.45 GHz and 5 GHz. The choice of the proper frequency value depends on the region of the world. In Europe, RFID devices work at 865–870 MHz under the protocol defined by ISO/IEC 18000-63 (EPC Class 1 Gen 2 [20]). The typical passive RFID transponder consists of the chip and the antenna (Figure 1a). Access to the chip internal memory can only be achieved by a read/write device (RWD) using the radio interface [21].

Another group of growing interest contains semi-passive RFID transponders equipped with built-in an extra supply source (e.g., lithium battery which are exchangeable or not), (Figure 1b). Generally, additional power obtained from the battery is used to enlarge the integration zone (IZ) [21], but it can also be used to power supplementary functions implemented in tags (Figure 1c), such as measurements of physical quantities (temperature, humidity, pressure etc.), storing gathered data in extended memory or exchanging information via serial interface.

Regardless of the frequency band as well as the above mentioned class of UHF RFID tags, the classically understood RFID transponder is a combination of an antenna and a chip [21]. A known and commonly used method of manufacturing such a device is its implementation in the form of a unitary structure in which the chip is, e.g., glued [22], soldered [23], or otherwise connected [24] to the antenna terminals. In such a design of the transponder, the antenna is made of conductive materials on a rigid (glass-epoxy [25], ceramic [26], etc.) or flexible (Kapton by DuPont, Wilmington, DE, USA [27], PET [28], paper [29], et al.) substrate carrier. The target product addressed to the consumers of RFID identification systems is enclosed in housing and protected against environmental conditions. According to contemporary conception of automatic identification RFID systems, transponders are fixed to objects permanently or temporarily [21]. It causes, that tags are manufactured in various shapes, e.g., as a label, a card, a glass tube, a disc, or a coin, as well as other housing with plastic, glass, metal, ceramic, etc. Nevertheless, in spite of existing belief, a universal design of transponders intended for marking any object does not exist. In any case of RFID implementation, the tag should be selected or (which is more advantageous) designed for a given object, taking into account many conditions of its operation in the target application of RFID system.

The authors’ approach to design the RFID transponder is shown in Figure 1d. It is based on the invention revealed in the patent “Textronic RFID transponder” (Section 5). In the proposed structure, the antenna and chip are located on physically separated and galvanically isolated substrates. The antenna module can be fabricated by embroidery or sewing with conductive threads as well as by other techniques, e.g., by pressing metal wires into the textile material. The microelectronic module can be manufactured as a semi-product (e.g., button or tab) and then it can be integrated by sewing with the labelled textile object. It may consist of a typical printed circuit board (PCB, or other rigid substrate used in the electronic technology) to which the RFID chip is attached. In most cases known from the literature of the typical transponders [30], the chip is bounded straight to the antenna terminals by soldering or using special adhesives. Further, in the presented conception, these typical processes can be used but only to assembly the microelectronic module. Then the resulting semi-product can be attached to the fabric in ways used in the textile industry. Thanks to such a conception it is possible to avoid problems with connecting the RFID chip to a flexible and ragged textile substrate, and thus to avoid many problems with protecting the semiconductor integrated circuit (IC) [31,32,33]. Since both parts are joined with the coupled inductive circuits, the problem that has to be overcome is to achieve the impedance matching between the inductive components. It can be solved by using a so-called parasitic loop (Section 2.2). Therefore, one of the main goals in the paper is to focus on the process of designing the method of matching the impedance of the antenna and the chip that are separated by the coupling system (Figure 1d).

### 1.3. State of the Art 

The classically understood antenna system that includes the radiator terminated with pads to which the chip is connected describes the construction of each passive RFID transponder (Figure 1a–c). In the concept of the proposed RFIDtex structure, the antenna, and the chip pads are galvanically separated between independently and differently manufactured modules (Figure 1d), in which the inductive coupling circuits (characterized by the mutual inductance *M_TAC_*) ensures the correct operation of the tag. It is known that there are designs of passive RFID tags in which the coupling inductive loops are sometimes used between the radiator and the chip [34]. However, both components are always integrated on a one common rigid or flexible substrate and the main reason for implementing such a solution is only to ensure the correctness of the impedance matching. In this context, the proposed separation of the antenna and microelectronic modules is unconventional in the literature on the subject. It should be also emphasized that despite the initial difficulties anticipated in future implementations of the design concept, the RFIDtex idea will provide a possibility of manufacturing textronic structures (i.e., the production of the antenna module and its integration with the microelectronic module made as a semi-product) directly in the textile industry.

The subject of antennas integrated with textile materials is the area of intense interest of research centers around the world. In most cases, preliminary usable structures of 50 Ω dedicated to antennas for classic radiocommunication systems are considered in scientific publications or design studies. Some of them concern single- [35,36,37,38] or multi-band [39,40] constructions, aimed at unlicensed Industrial–Scientific–Medical (ISM) bands [41,42]. Typically, these constructions are made by combining: (a) classic conductive materials with a textile base (e.g., copper on felt [35], leather [41], and other bases [42]) or (b) innovative conductive textile materials with classic textiles (i.e., with denim [39] and others [42]). Integration of typical flexible antennas with textiles, which are made, for example, on PET films is also more and more common in practical designs [40,42]. Due to the fact that numerically controlled machines are becoming more and more available, the literature reports also include structures sewn or embroidered with conductive threads [37,38].

In the area of RFID technology, the first laboratory designs of sewn/embroidered passive transponder antennas dedicated executively to the UHF band can be encountered in a few publications of Finnish–American research teams [43,44,45]. The classic connection of the chip and the antenna are reported in these works. Although RFID tags are made on a flexible substrate, such a concept by definition constitutes an obstacle to the practical use of these devices in the expected life cycle of textile products. Thus, the interest in the discussed 50 Ω textile antennas dedicated to classical radiocommunication systems, noticed in recent years, does not translate into R&D works in the field of the RFID technology, which could contribute to dissemination of cheap and useful transponders for the IoT sector. This is primarily due to the fact that both the operation principles of the RFID devices and the issues of determining their parameters are different than means known in the theory of classical radio systems. It causes an increased risk of applying the considered devices. However, simultaneous development of the proposed RFIDtex concept with the definition and verification of the RFIDtex tag parameters that significantly influence the process of synthesizing the interrogation zone (IZ) [21,46,47,48] guarantee market success in this respect.

A very interesting work in the subject matter was published in 2020 [49]. The authors analyze constructions of the textile tags with the inductive coupling, used in the laundry industry. The chip is bounded to the inductive loop made on the PCB substrate, and then the transponder is attached to the antenna with adhesives materials. The tag produced in this way is intended to be attached to the labeled product in a separate logistic process when it is prepared for entry into the automatic identification laundry system. In the paper, several different modifications are proposed and compared with commercial products.

To sum up, although textile dipole antennas dedicated for RFID transponders are known from the scientific papers, in all considered designs, the chip is bounded straight to the antenna terminals by soldering or using special adhesives. However, there is a group of tags dedicated to the logistics of laundry, but it is still not manufactured directly on the marked textile product at the time of its production. In the design proposed in this paper the antenna and chip are located on physically separated and galvanically isolated substrates—the antenna with the coupling circuit is sewn on a textile substrate, whereas the chip is soldered to the coupling circuit made on the PCB; both parts of the transponder are assembled by sewing with non-conductive threads without galvanic junctions.

## 2. Materials and Methods

### 2.1. Impedance Matching

**Figure 2:**
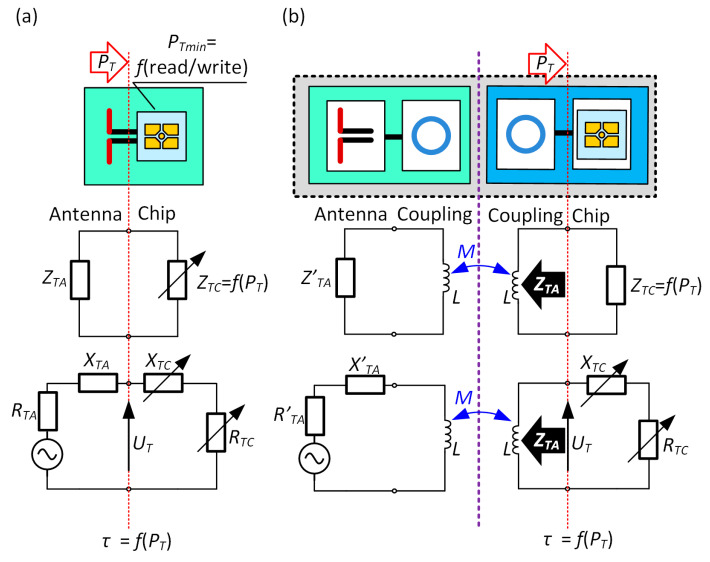
Electrical equivalent diagram of antenna connected to radio frequency identification (RFID) chip: (**a**) Typical passive transponder; (**b**) Transponder with coupling system (including antenna and chip coupling circuits).

One of the most important things in the process of designing the RFID transponder is the impedance matching between the antenna and the input circuit of the chip attached to antenna connectors. In order to avoid signal reflections in the transmission path the impedances of the antenna *Z_TA_* and the chip *Z_TC_* have to be equal *Z_TA_* = *Z_TC_* * and have to show opposite nature of the reactance (Figure 2) [50]. Then, the power transfer coefficient *τ* expressed by the formula:

(1) $$\tau =\frac{4Re(Z_{TA})Re(Z_{TC})}{\left(Re(Z_{TA}+Z_{TC}\right){)}^{2}+\left(Im(Z_{TA}+Z_{TC}\right){)}^{2}}.$$

describes the quality of the impedance matching and is equal *τ* = 1.

In the passive or semi-passive UHF transponders, the stability of the *Z_TC_* parameter constitutes a significant problem. Its value changes during the chip operation, depending on the strength of the electromagnetic field and thus influences the amount and efficiency of gathered energy. In consequence, the power *P_T_* harvested in the radio frequency (RF) front-end of the chip as well as impedance *Z_TC_* varies with tag orientation and location relative to the plane of the RWD antenna. In this way, they are also related to set communication protocol parameters, environmental disturbances, as well as the activity performed in digital circuits (power supply demand). Thus, in order to meet the impedance matching requirement, the antenna has to be designed with respect to the known operating point of the input characteristic of the chip. Moreover, the need of the greatest possible read range [21] should be taken into consideration. This means that the *Z_TC_* value corresponding to the minimal power *P_Tmin_* (chip sensitivity) has to be assumed in the antenna synthesize process as the impedance conjugated to *Z_TA_*. Both parameters are specified in the manufacturers’ datasheets. The *P_Tmin_* value depends on the type of the chip: used IC technology, design, and efficiency of the front-end or load generated by the internal circuitry. The input resistance *R_TC_* (at *P_Tmin_*) of the typical chip is equal a few to tens of Ohms and the reactance *X_TC_*—several hundreds of Ohms. Both parameters depend on the design of the electronic circuit used in the RF front-end.

### 2.2. Antenna Construction for Textile UHF RFID Transponder with Coupling System

**Figure 3:**
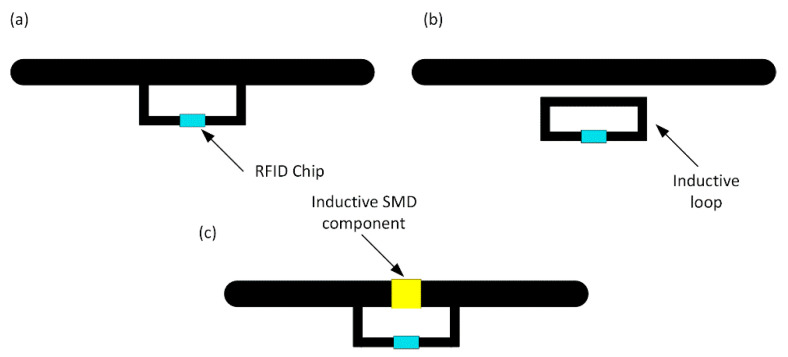
Means of impedance matching in RFID transponder: (**a**) T-match; (**b**) Parasitic loop; (**c**) Inductive surface mounted (SMD) component.

There are several methods of the impedance matching with uncomplicated structures that could be implemented in RFID systems operating over a wide frequency range of the UHF band (860–960 MHz). A conductive element such as a T-matching (Figure 3a) or a parasitic loop (Figure 3b) attached to the radiator is the most frequently used to adjust the antenna impedance. Sometimes it is easier to use discrete surface mounted inductors (SMD—surface mounted device) [51], especially at development stage of a designed device (Figure 3c). These matching methods cannot be used with any RFID IC.

**Figure 4:**
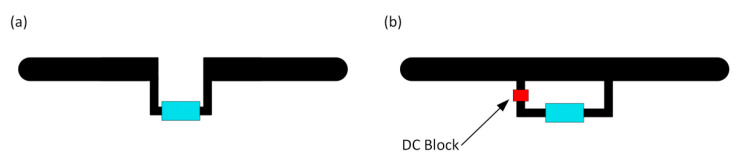
Transponder antenna: (**a**) Antenna with open arms; (**b**) Antenna with shorted arms.

An example is the AMS SL900A (AMS AG, Premstaetten, Austria) [52] semi-passive chip which is equipped with a system for gathering energy from the electromagnetic field. The internal energy harvester is joined inside semiconductor structure to the antenna connectors. For this reason, the antenna with an impedance matching circuit in the form of the shorted loop attached to the AMS SL900A RF input blocks the proper operation of its internal power supply. In such a case, it is necessary to use a structure with separated arms (Figure 4a), tuned using micro-strip lines or inductive SMD elements. It causes a number of problems during the design process—for example, it is sensitive to harsh environmental conditions as well as to even small changes in geometrical dimensions of the antenna radiator (i.e., dimensions of antenna arms).

Since the impedance matching in the form of T-match or parasitic loop is the best option after all, the problem of short circuit of the energy harvester input has to be solved. It can be done by introducing a capacitor with a properly selected capacity that operates as a DC Block (Figure 4b). It blocks the direct current flow which enables the harvester to work, but its impedance is negligibly small in the frequency of the UHF band. Thus, the DC block can be neglect when the antenna design is being developed in numerical calculations.

**Figure 5:**
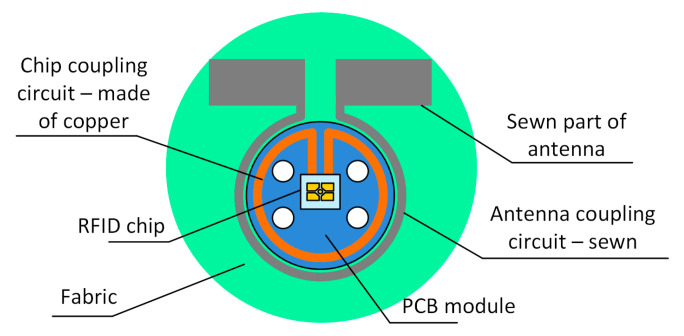
Coupling system between RFID chip and sewn antenna used in developed textronic transponder.

It should be noted that the described impedance matching methods are applied primarily to antennas fabricated in the PCB technology; although, the antenna manufactured on a flexible textile substrate is considered in the publication [53]. However, due to the use of the special coupling system (Figure 1d), a new approach to the problem has to be performed in the case of the presented antenna made by sewing with the conductive thread. The proposed coupling system allows the authors to avoid using the strap [54] when assembling the RFID chip, but it has to be taken into consideration when designing the impedance matching circuit (Figure 5).

The idea of impedance matching by the parasitic loop (Figure 3b) is used in the RFIDtex transponder. The microelectronic module (Figure 1b) composed of the chip and its coupling circuit is placed on a separated substrate (e.g., PCB). The correct impedance matching is achieved thanks to mutual inductance *M_TAC_* between the parasitic loop and the antenna coupling circuit, despite the existence of galvanic separation of both modules. As a consequence, it gives high enough complex impedance value seen at the IC terminals. Since the chip module is manufactured in the form of a stand-alone product, e.g., button or label, it can be prepared to operate in harsh environmental conditions. On the other hand, it can be suited to be attached to the sewn antenna by means that are typical for the textile industry. For example, the microelectronic button can be attached to the textile part using classical non-conductive threads. Hence, the natural flexibility of the tags created in this way and their susceptibility to use in all subsequent logistic processes. It improves the comfort of creating the object identification system and reduces implementation costs as useful throughout the entire product life cycle [19].

### 2.3. Design Assumptions and Numerical Calculations

The main goal of the research work was to synthesize the fully functional textile RFID transponder. Conducting numerical calculation, the authors focused on achieving the impedance *Z_TA_* at the level of a dozen Ohms in the real part and several hundred Ohms in the imaginary part, seen at the chip terminals. It was assumed to keep the antenna small, with compact geometric dimensions. The process of antenna design for the textronic UHF RFID transponder was performed on the basis of numerical calculations conducted in the EMCoS Antenna VLab software. The investigation was applied to the commercially available AMS SL900A RFID passive/semi-passive chip (in QFN16 package). A single conductive thread to embroider the shape of antenna and coupling system on a textile substrate was chosen to fabricate the embodiment. Moreover, it was assumed that the designed antenna should have omnidirectional radiation pattern (typical dipole antenna), relatively small geometrical size and a few hundred Ohms of imaginary part of the impedance *Z_TA_* in the frequency band from 800 MHz to 1 GHz (which cover the UHF RFID band of 860–960 MHz, which makes the RFIDtex tag usable all over the world).

The measured values of the chip impedance *Z_TC_* are presented in Table 1. Since the AMS SL900A can operate in the passive and semi-passive mode, the impedance is determined for both cases at the measured sensitivity *P_Tmin_* [25,55].

**Figure 6:**
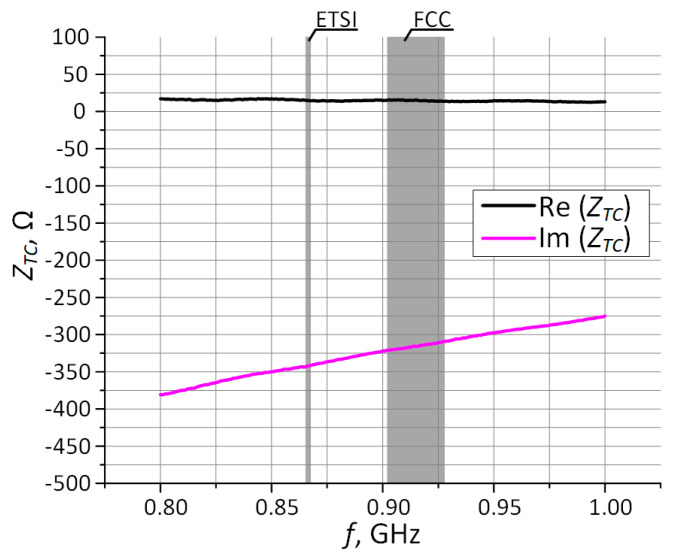
Measured characteristic of AMS SL900A chip impedance.

The knowledge of the chip impedance exact value at a given frequency is crucial for defining matching efficiency as determined by the coefficient *τ*. It should be noticed that the considered chip has the extremely large imaginary part of the impedance. This is a major problem, especially when the impedance matching is searched for coupling between sewn wires and milled PCB tracks. Considering the research conducted in [25] and the results in Table 1, the practical conclusions can be drawn that the same design of antenna can be implemented in both passive and semi-passive transponders, because the parameter *P_Tmin_* is almost constant whether or not supplementary source is used. Therefore, the AMS SL900A chip impedance characteristic as a function of frequency is determined for the sensitivity *P_Tmin_* = −15 dBm (Figure 6). This value represents a compromise for passive or semi-passive mode in the frequency band of 860–960 MHz [55].

**Figure 7:**
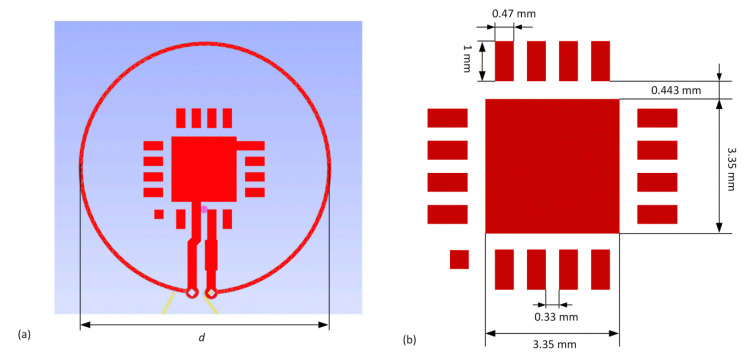
Microelectronic module of antenna made: (**a**) numerical model in EMCoS Antenna VLab software, diameter *d* = 13 mm; (**b**) imported footprint of QFN16 AMS SL900A.

Dimensions of the antenna depend primarily on the resonance frequency *f*_0_ and the relative permittivity *ε_r_* of the dielectric layer on which the construction is manufactured. In the case under consideration, the antenna design was prepared taking into account two different dielectric substrates implemented in both modules of the developed RFIDtex tag. The microelectronic module was made on low-loss double sided laminate ISOLA FR408 (ISOLA GmbH, Düren, Germany), (thickness of dielectric layer *h* = 0.51 mm, thickness of copper layer 18 µm, *ε_r_* = 4.44, tg*δ* = 0.0096). Its parameters were determined at the frequency of 1.1 GHz in the special test bed consisted of the VNA Keysight PNA-X N5242A (Keysight, Santa Rosa, CA, USA) and the QWED split post dielectric resonator (SPDR), (QWED Sp. z o.o., Warsaw, Poland). The appearance of the antenna pattern on the chip side is shown in Figure 7a. It includes the footprint of the AMS SL900A chip (Figure 7b) and the coupling circuit. The module was made in the form of a button (that can be treated as a semi-product in the industry) and its dimensions were determined mainly by the QFN16 housing. The PCB was designed in a specialized electronic computer-aided design (CAD) system. Then, the inductive loop of the coupling system was fitted in the way to obtain the smallest possible size of the module but also to leave a place for four mounting holes and to keep a minimum possibility of expanding the electronic circuit with additional discrete elements and connections (AMS SL900A is equipped with SPI interface, input of 10-bit A/D, etc.). Such a design derives from the idea of further research towards the RFIDtex sensors, which will be the subject of next publications. The PCB pattern was saved in the Gerber files as an open ASCII vector format for PCB manufacturing devices. In general, the proposed model can be easily redesigned for various types of RFID chips. However, if a smaller button was considered, then it might turn out that two turns of inductive loop in coupling system were required for establishing the proper impedance matching. The Gerber design was also imported to the EMCoS Antenna VLab software in order to develop the antenna module of the RFIDtex transponder.

**Figure 8:**
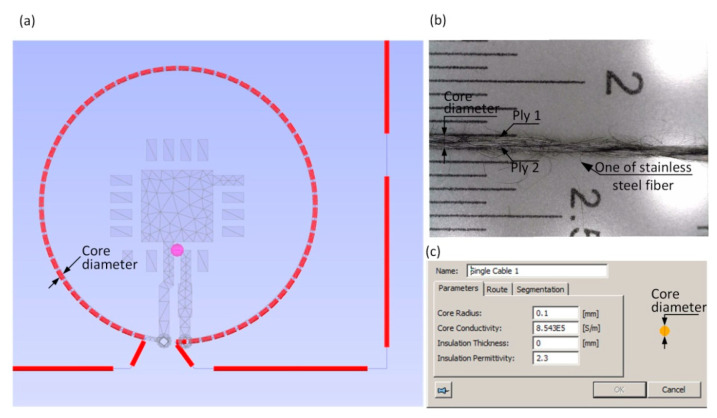
Definition of antenna module of the UHF RFIDtex transponder: (**a**) Numerical model; (**b**) Conductive Adafruit 2 ply stainless thin thread; (**c**) Input data on conductive thread properties.

The selected textile substrate used to design the antenna module is a combination of linen fabric and fleece. The complex permittivity of the fabric was measured at the frequency of 1 GHz that is close to the operating band of the designed UHF RFID transponder. It was determined in the laboratory stand composed of Compass Technology Epsilometer (Compass Technology Group LLC, Alpharetta, GA, USA) with integrated Copper Mountain R60 1-Port 6 GHz Vector Network Analyzer (Copper Mountain Technologies, New York, NY, USA) [56]. The relative permittivity is equal *ε_r_* = 1.03 and the dielectric loss tg*δ* = 0.01 at *f*_0_ = 1 GHz (thickness of fabric: 400 µm). The parameters of Adafruit 640-2 ply stainless thin thread (Adafruit Industries, New York, NY, USA) (Figure 8) was used in antenna numerical calculations (conductivity 8.54 × 10^5^ S/m and diameter 0.2 mm obtained on the basis of measurements carried out for the selected section of the conductive thread). It should be noticed that the calculated conductivity of Adafruit 640 thread is 67 times less than that of copper, which makes it very difficult to match the chip and antenna impedance in comparison with, e.g., PCB transponders.

**Figure 9:**
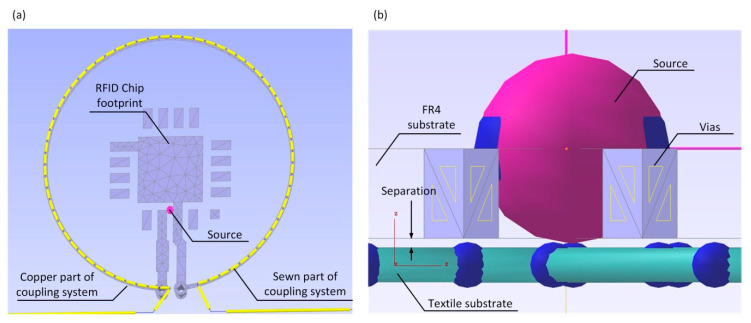
Idea of coupling system in Textronic UHF RFID Transponder: (**a**) Top view of the model; (**b**) Cross section view of the model.

The numerical model of the antenna was prepared in the EMCoS Antenna VLab software and calculated using TriD solver. The main challenge during the antenna design process was to develop a model of the working coupling system. The significant problem was to determine the distance between the PCB substrate and the flexible textile substrate (Figure 9). Some simplifications had to be assumed due to the fact that the button was covered with a layer of varnish and placed directly on the rough textile material. The separation between the copper circuit on the button and the conductive thread was established as 150 µm.

**Figure 10:**
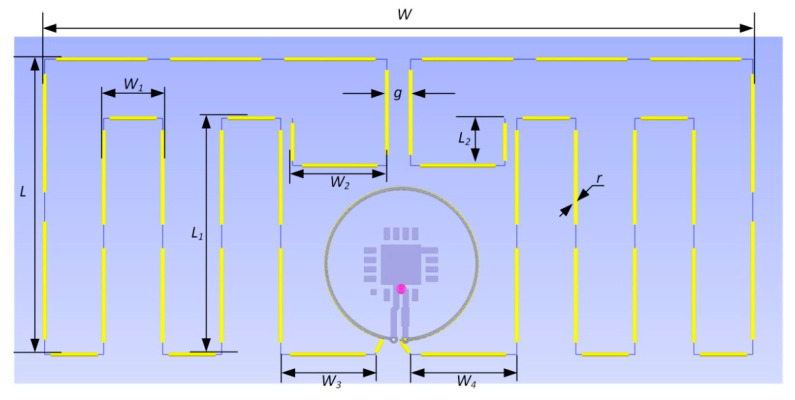
Design of transponder antenna by using EMCoS Antenna VLab elaborated model: *W* = 60 mm, *L =* 25 mm, *W_1_* = 5 mm, *L_1_* = 20 mm, *W_2_* = 8 mm, *L_2_* = 4 mm, *g* = 2 mm, *d* = 13 mm, *r* = 0.1 mm, *W_3_* = 8 mm, *W_4_* = 9 mm.

A lot of different cases were taken into consideration during the design process. The final antenna model is shown in Figure 10. Its shape is a classic meandered dipole. Due to the fact that the transponder should be relatively small, the method in which the legs of the dipole are drawn as meanders was used. It allowed to obtain the required length of the dipole arms, while reducing the area of the antenna. The shape of the antenna resulted from a series of simulations on the basis of which the best impedance matching was reached. It was a compromise between the number of contained meanders and the geometric size of the transponder antenna.

The results confirm the validity of assumptions made for the proposed antenna of textile UHF RFID transponder with the coupling system.

## 3. Results

### 3.1. RFID Transponder Manufacturing

**Figure 11:**
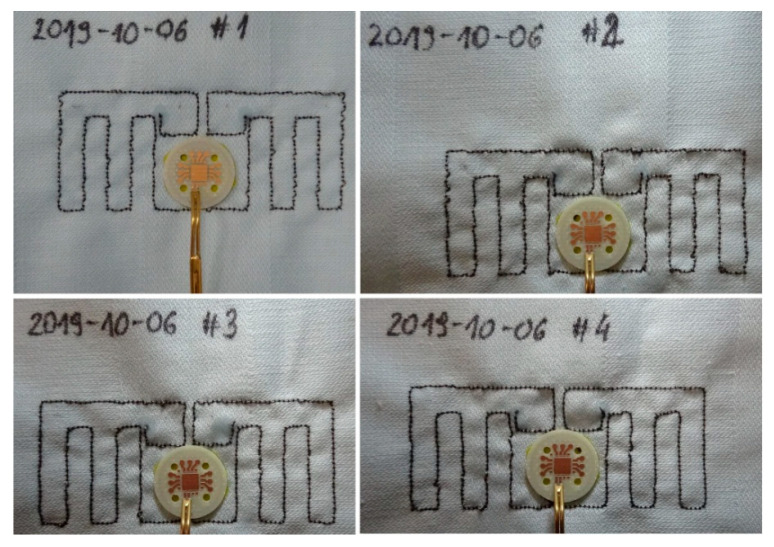
Test samples of RFIDtex transponder manufactured on the basis of EMCoS Antenna VLab model.

**Figure 12:**
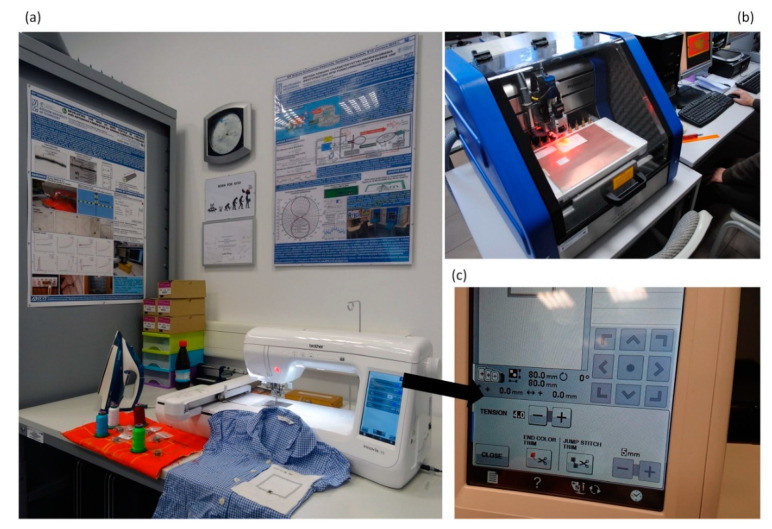
The manufacturing stand: (**a**) Computer controlled sewing process; (**b**) printed circuit board (PCB) plotter LPKF ProtoMat S100; (**c**) Embroidery machine Brother INNOV—IS V3.

Test samples of the elaborated RFIDtex transponder were made on the basis of the obtained numerical model (Figure 11). The antenna module of samples was manufactured on substrates that combine linen fabric and fleece, by using embroidery machine Brother INNOV-IS V3 (Brother Industries, Nagoya, Japan). In addition, the chip module was milled (footprint for the AMS SL900A chip) by using PCB plotter LPKF ProtoMat S100 (LPKF Laser & Electronics AG, Garbsen, Germany), (Figure 12).

### 3.2. RFID Antenna Impedance Measurements

**Figure 13:**
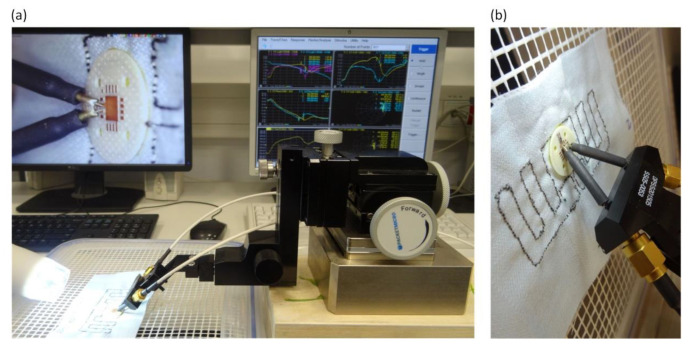
Measurement process of prepared samples: (**a**) Laboratory stand; (**b**) Probe.

The model calculations and corresponding measurements were carried out for the impedance matching at the frequency *f*_0_ = 866 MHz. The impedance parameters of the antenna were measured by using differential method [57] in which the VNA Keysight PNA-X N5242A and special probe (PacketMicro DPSS201505 SS05-0053 calibrated together with the JYEBAO RG316 cables) were applied (Figure 13).

**Figure 14:**
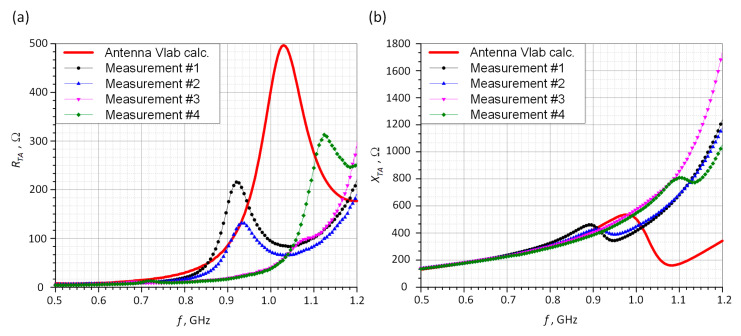
Antenna impedance: (**a**) Resistance; (**b**) Reactance.

**Figure 15:**
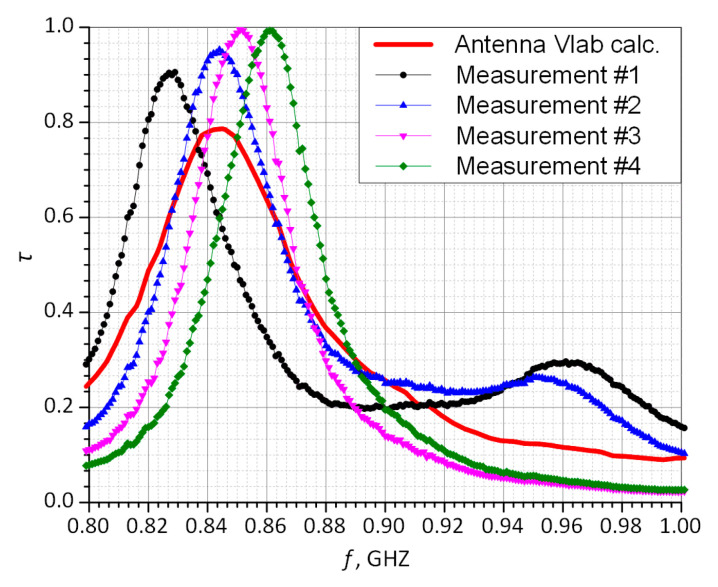
Power transfer coefficient vs. frequency.

The results obtained for the antenna impedance are summarized in Figure 14. The calculations and measurements carried out for the power coefficient are presented in Figure 15. The coefficient *τ* is calculated on the basis of (1).

In the presented design project, it can be seen that for the four sewn samples, a relatively large convergence of the impedance values at *f*_0_ operating frequency was achieved compared to the calculations. Antennas of number #3 and #4 are the samples for which the measured impedance differs the most from that in the simulation. The discrepancies result mainly from the established embroidery machine settings (Figure 12). The samples of number #1 and #2 were sewn at tension equal 1, #3: tension = 1.6, and #4: tension = 2.

For the prepared samples, the measured maximum values of power transfer coefficient are higher than that obtained with the numerical calculations of about 0.2. The parameter is close to one for samples #3 and #4 and is approximately equal to 0.9 for #1 and #2. At the resonance frequency of *f*_0_ = 866 MHz, the values of *τ* are as follows: #1: *τ* = 0.28, #2: *τ* = 0.51, #3: *τ* = 0.62, and #4: *τ* = 0.89. It can be easily seen that for each of the samples, the measured curves of power transfer coefficient are slightly different from the calculated results. However, from the practical point of view, the achieved *τ* values are satisfactory and the antennas can be implemented in the RFIDtex transponders. It should also be borne in mind that when assessing the quality of the impedance matching, the convergence of the real and imaginary parts of the antenna (resonance) as well as the *τ* coefficient should be taken into consideration. The discrepancies in the results were caused by different parameter setup of the embroidery machine. The each of the antennas was manufactured with various value of thread tension, what influences total length of the conducting path and in consequence its electrical parameters. Nevertheless, obtained impedance values prove that the fabricated textronic tag can operate properly in a real RFID system. The new approach to idea of impedance matching between the chip and the antenna was proven to be correct. Thanks to the coupling system, it is possible to simply place the chip on the PCB (e.g., made in the form of a button) and use classic chip connection methods such as soldering. The rest of the tag including the embroidered antenna can be easily manufactured in production lines of the textile industry.

### 3.3. Experiment Setup

The prepared antenna modules were used to build textronic transponders (TUT—transponder under test) based on the AMS SL900A RFID chip. Aside the AMS SL900A IC, the 56 pF capacitor used as the DC Block was attached to the PCB chip module. The transponder with the antenna of No. 2019-10-06 #1 (Figure 11) was used in the next stage of the investigations, in which different parameters of the elaborated devices were measured. First of all the threshold values of power passed in communication link were determined (threshold measurements). On the basis of results the read ranges of TUT in an exemplary RFID application were calculated. In addition, the radiation pattern diagrams of TUT were measured (rotation measurements—Section 3.4).

**Figure 16:**
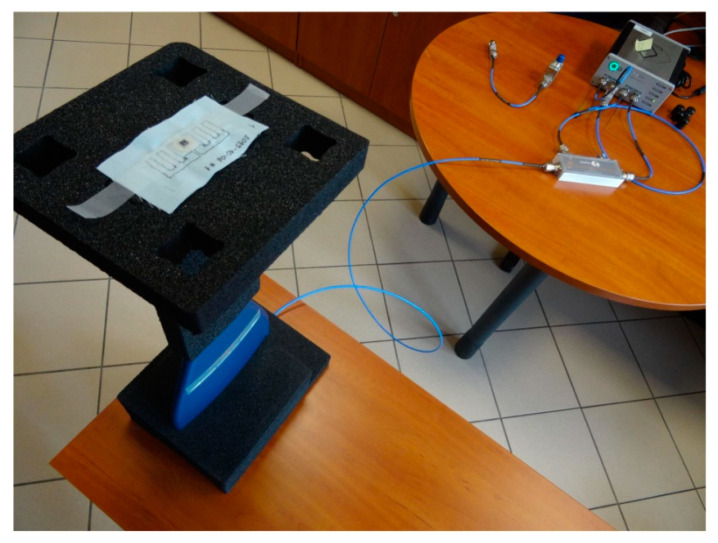
Test stand for measure of the boundary parameters of energy and data transmission.

Experiments were made using the Tagformance Pro Measurement System (Figure 16) with the Tagformance UHF v.12.3.2 software (Voyantic Ltd., Espoo, Finland), as well as a set of RF equipment including: 50 Ω directional coupler (frequency range of 600–1300 MHz, insertion loss TX to ANT 1.2 dB, insertion loss ANT to RX 6.5 dB), log periodic broadband Yagi antenna—Aaronia HyperLOG 7025 (Aaronia AG, Euscheid, Germany), (frequency range: 700 MHz–2.5 GHz, *VSWR* < 2, typical gain 4 dBi), set of solid PTFE flexible cable assemblies (Florida RF Labs, Stuart, FL, USA), and others RF components. The parameters of the ISO/IEC 18000-63 communication protocol (conforming to EPC Class 1 Gen 2 [19]) set during measurements are shown in Table 2.

The proposed structure of the textronic transponder were considered in aspect of usability to RFID applications that are compliant with the requirements of European Telecommunications Standards Institute (European version: ETSI EN 302 208, frequency band 865.6–867.6 MHz, 2 W ERP) as well as Federal Communications Commission (American version: FCC Part 15.247, frequency band 902–928 MHz, 4 W *EIRP*, 1 W of transmitter output power with maximal gain of 6 dBi). The test stand was properly calibrated by using Voyantic wideband UHF reference tag v1 in order to compensate reflections in the test room that is non-anechoic. For this purpose, the free-space path loss (FSPL) measurement was performed.

In the experiment, the Tagformance Pro as the RWD sends information to TUT by modulating an RF carrier using double-sideband amplitude shift keying (DSB-ASK) with a pulse-interval encoding (PIE) format (forward link). The TUT receives their operating energy from this modulated RF carrier. The Tagformance Pro receives information from the TUT by transmitting an unmodulated RF carrier and listening for a backscattered reply (return link). The TUT replies in backscatter communication by modulating the amplitude of the RF carrier. The data sent to Tagformance Pro in response to commands are encoded in FM0 modulated subcarrier format.

### 3.4. Threshold Measurements

Measurements of the limit parameters of energy and data transmission were performed in the 800–1000 MHz band. In the experiment the power on tag forward (*P_Pwr_*) and the power on tag reverse (*P_Btr_*) were determined.

The first parameter is defined as the minimum power needed to properly start (power up) the tested transponder, send the Query command, and obtain a TUT response. It can be written as

(2) $$P_{Pwr}=P_{EIRP}{\left(\frac{c}{4\pi fr}\right)}^{2}=P_{R}\left(\mathrm{dBm}\right)-L_{R}\left(\mathrm{dB}\right)+G_{R}\left(\mathrm{dBi}\right)-FSPL\left(\mathrm{dB}\right),$$

where *P_EIRP_* means the effective isotropic radiated power, the speed of light *c* = 2.998 × 10^8^ m/s, *f* is frequency, *r*—distance between TUT and RWD antenna, *P_R_*—the RWD output power, *L_R_*—RWD antenna cable loss, and *G_R_* denotes the gain of the RWD antenna.

With regard to the correct supply of TUT, the maximum operating read range *r_PwrMax_* (forward link) in an exemplary RFID system can be determined on the basis of power limitation, according to the equitation:

(3) $$r_{PwrMax}=\frac{c}{4\pi f}\sqrt{\frac{P_{EIRPmax}}{P_{Pwr}}},$$

where *P_EIRPmax_* means the maximal effective isotropic radiated power. For ETSI EN 302 208 standard, *P_EIRPmax_* = 3.28 W *EIRP* (35.16 dBm), which corresponds 2 W ERP (33 dBm). In the case of FCC Part 15.247, *P_EIRPmax_* = 4 W *EIRP* (36 dBm).

The power on tag reverse (*P_Btr_*) can be defined as the effective backscatter transmit power (*EIRP*) of the TUT when answering to an RWD command. As before, with regard to this parameter, the maximum operating read range *r_BtrMax_* (return link) of an exemplary RFID system can be determined according to the relationship:

(4) $$r_{BtrMax}=\frac{c}{4\pi f}\sqrt{\frac{P_{Btr}}{P_{Rmin}}},$$

where *P_Rmin_* means the RWD sensitivity. As per requirements of EPCglobal, *P_Rmin_* = 40 pW (−74 dBm), which means receiver operation with sensitivity of −70 dBm with 4 dBi gain of antenna, configuring the transmission system at *P_EIRPmax_*.

**Figure 17:**
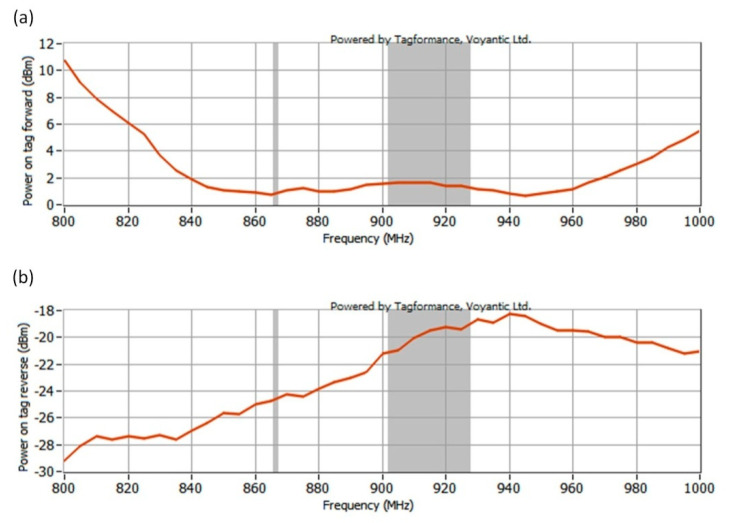
Graph obtained by Tagformance (Voyantic) showing: (**a**) Power on tag forward; (**b**) Power on tag reverse.

The measured curves of power on tag forward (*P_Pwr_*) and the power on tag reverse (*P_Btr_*) are presented in Figure 17.

**Figure 18:**
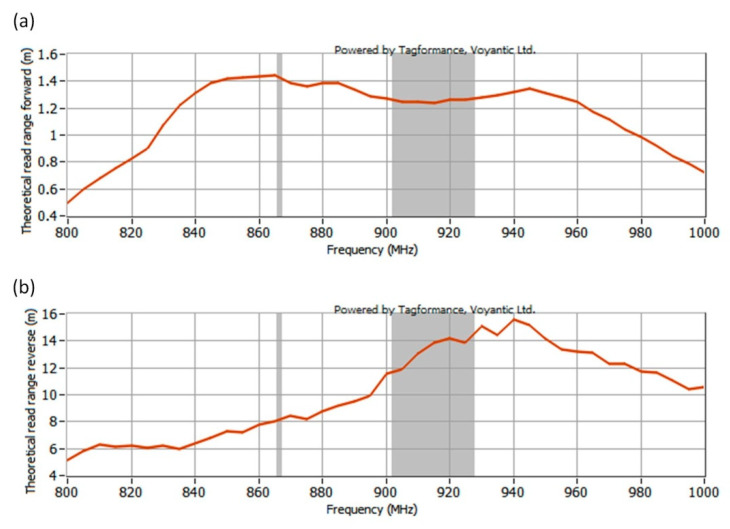
Graph obtained by Tagformance (Voyantic) showing: (**a**) Read range-forward link; (**b**) Read range-return link.

The read range (forward link) *r_PwrMax_* and read range (return link) *r_BtrMax_* were determined on the basis of power measurements, assuming the operation of the tested transponder in a system compliant with ETSI EN 302 208 (*P_EIRPmax_* = 3.28 W *EIRP*). The calculated read range is shown in Figure 18.

### 3.5. Rotation Measurements

**Figure 19:**
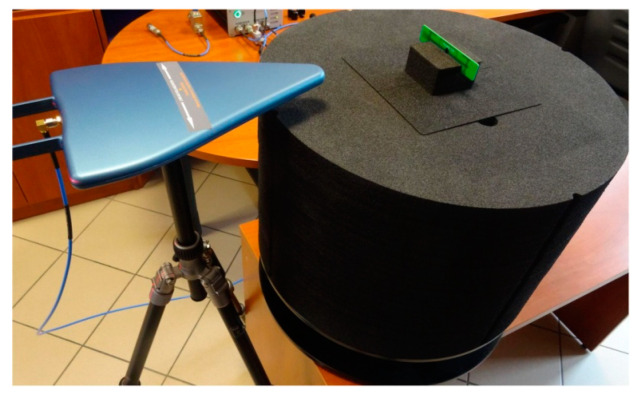
Test stand for radiation pattern determination, in calibration setup.

Measurements of the radiation pattern were made for the textronic transponder #1 by using the Voyantic stand-alone tag rotation system (TF-AC-ROT). The system was located in an arranged free space of the test room, 35 cm from the AARONIA HyperLOG 7025 antenna. Prior the research, the stand was re-calibrated by using Voyantic wideband UHF reference tag v1 to compensate the reflections in the test room that is non-anechoic (Figure 19).

**Figure 20:**
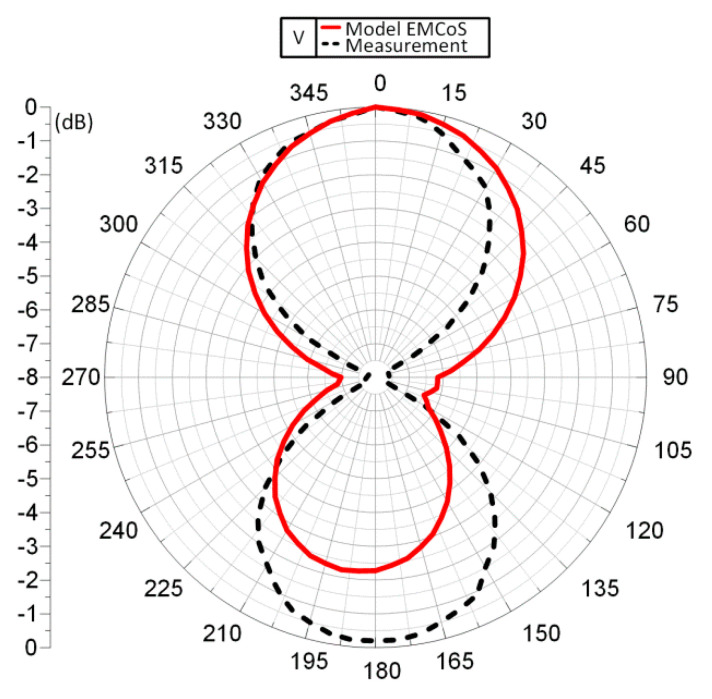
Diagram of antenna radiation pattern at 866 MHz.

The measurement of the horizontal radiation pattern diagram (Figure 20) was performed for frequency of 866 MHz, angle step of 5°, TX power step of 0.1 dB.

**Figure 21:**
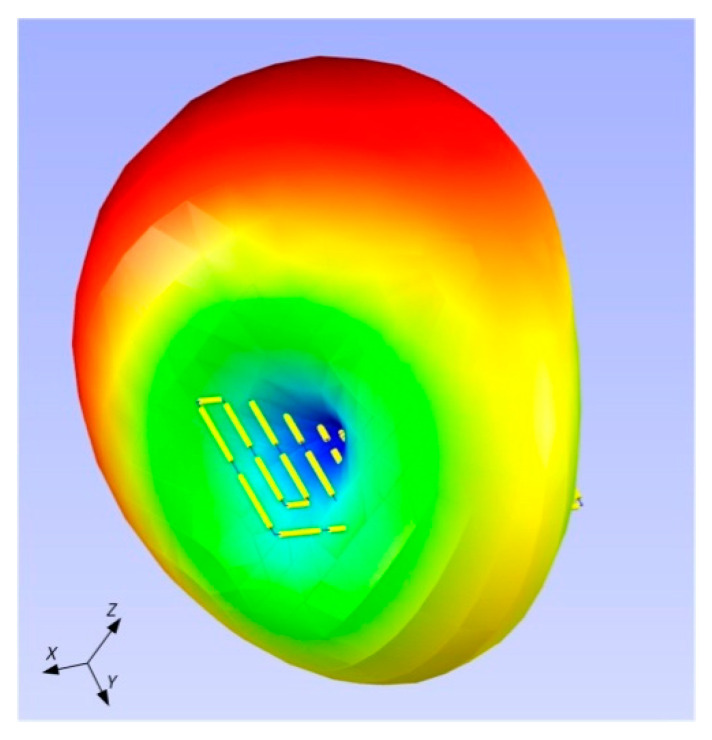
3D view of calculated radiation pattern for 866 MHz.

**Figure 22:**
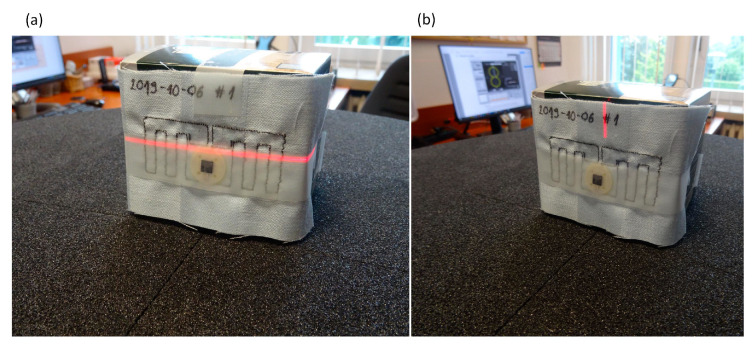
Measurements of TUT radiation pattern; precise positioning of test transponder: (**a**) horizontal axis (**b**) vertical axis.

It can be noticed that the calculated radiation pattern slightly differs from the shape typical for a classic dipole antenna. As a result, there is a difference in the shape of the characteristic between the calculated and the measured one (the measured radiation pattern has the shape typical for dipole antenna). On the three-dimensional radiation pattern calculated and generated in EMCoS Antenna VLab (Figure 21) it can be observed that the beam is slightly directional and not symmetrical. The aforementioned distance of the PCB (made in the form of a button) from the sewn antenna should be taken into account. This may have an effect on the coupling system, making the radiation pattern slightly different to that measured. Moreover, the measurements were not made under the ideal conditions (as opposed to the ideal simulation conditions). The process of measuring the radiation pattern of the textronic transponder on the specially prepared stand is presented in the Figure 22.

**Figure 23:**
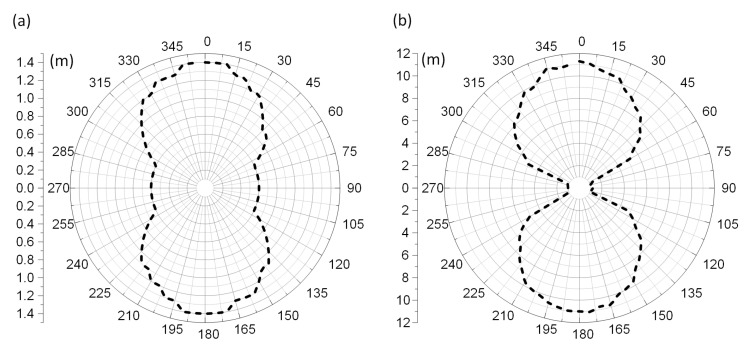
Diagram of transponder read range: (**a**) Forward link; (**b**) Return link.

The operating read range (Figure 23) was determined on the basis of Equations (2)–(4), assuming the TUT operation in a RFID system compliant with the requirements of ETSI EN 302 208 and EPCglobal: *P_EIRPmax_* = 3.28 W *EIRP* (35.16 dBm), *P_Rmi n_* = 40 pW (−74 dBm).

Despite different laboratory setup for the threshold and rotation measurements, the obtained results of the read range are consistent. It should be noted that the sample #1 has the worst *τ* coefficient and it gives the smallest (although acceptable) read range. The authors drew attention to the problem with performing precisely (repeatedly) the sewing process that would provide predictable geometric sizes of samples. Thread tension can affect the power transfer coefficient. Therefore, when the antenna is properly designed and efficient impedance matching is achieved, the biggest problem at the production site will be to maintain stable parameters of sewing machines. However, the obtained read range for the proposed sample #1 with the SL900A semi-passive chip gives an opportunity to develop the RFIDtex tag that meets all the requirements desired in applications pointed in Section 5.

The antenna and coupling system for the prepared microelectronic module are designed only with considering parameters of substrate materials, conductive tracks, and air as the surrounding environment. However, in all applications mentioned in Section 5, the RFIDtex tags are identify when the marked clothes are not being worn by users, thus the performed experiments should meet the desired expectations, especially according to the read range. Nevertheless, the problem of the antenna detuning by metal or liquids (including human body) remains pended and will be considered in the next stage of the research. For example, the impact of the human body on the RFIDtex tag performance is evident and definitely negative taking into consideration its dielectric parameters (*ε_r_* = 50, …, 60, tg*δ* = 1.2; water *ε_r_* = 80). Since the body influences the impedance of the antenna, it detunes the matching circuits.

## 4. Discussion and Conclusions

The research confirms the possibility of synthesizing the textronic RFID transponder that meets the assumptions claimed in the author’s patent (Section 5). The simulation and measurement results prove the concept of manufacturing a relatively small antenna in the form of a meandered dipole sewn in with a single thread, and further, that can be connected to the RFID chip through the coupling system without galvanic junctions. Moreover, effects of pattern sewn on a flexible linen fabric on the wireless performance of the passive RFIDtex tag were examined. The phenomenon of coupling between the chip placed on the PCB substrate and the antenna radiator made of conductive thread was investigated when searching for the problem under consideration. The idea of a coupling system between physically separated parts of the antenna was finally tested. Despite a slight shift in the maximum power transmission coefficient from the assumed resonance frequency, the achieved parameters of the tag clearly indicate that it can correctly communicate with the RWD. In addition, it should be noticed that the coupling between both transponder parts works properly and the impedance matching in this case is possible.

Since the chip module is fabricated as a semi-product it can be easily integrated with the labeled textile materials. The main advantage of the elaborated construction is its flexibility and environmental resistance, with the possibility of implementation in technological production lines that are normally used in the textiles industry. This approach reduces production costs and supports the design of automatic identification systems that can be applied at all subsequent stages of the product’s life cycle. On the basis of the prepared model other constructions of the RFIDtex transponder can be developed, for example designs with other RFID chips with a lower imaginary value of the impedance part. Future works can include a construction in the form of embroidered fully filled shapes and studies of the effects of reducing amount of used conductive thread. They can be based on the numerical model of the RFIDtex transponder that was prepared in the EMCoS Antenna VLab software.

Authors provide full information about the proposed construction that confirms the usefulness of the RFIDtex idea. The paper is a very good compendium of knowledge that can be used for developing further designs. Such a comprehensive studies on the UHF RFIDtex transponder antenna cannot be found in the literature on the subject. It contains:

- comprehensive parameterization of all materials (substrates and threads) with experimental verification at every stage;
- the possibility of simplifying wired model of conductive threads on the basis of performed investigations;
- full determination (numerical calculations and measurement) of parameters with their critical evaluation in the operational context, which is to lead to implementation in the industry.

## 5. Patent

**Figure 24:**
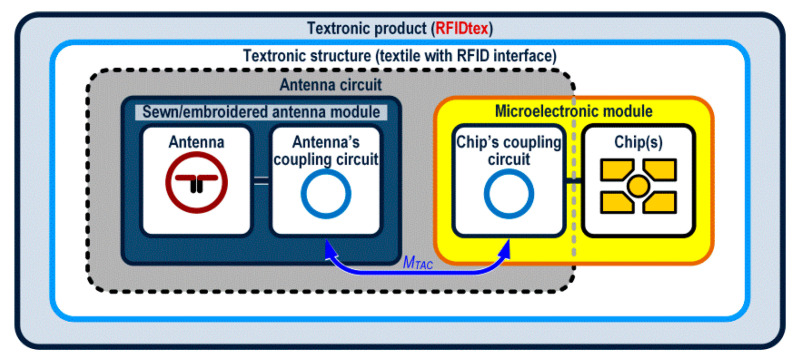
Idea of textronic structure with RFID interface (RFIDtex transponder) implemented in textronic product.

In the patent “Textronic RFID transponder” by Jankowski-Mihułowicz P., Węglarski M. (“Tekstroniczny identyfikator RFID”, Polish Patent Office, patent no: PL 231291 B1, granted on: 4 October 2018, property: Rzeszów University of Technology), the authors proposed the unique design of transponder with galvanically separated chip and antenna circuits. Since it is dedicated to integrate with textile product, it is called RFIDtex transponder (RFIDtex tag). In the RFIDtex tag concept, the coupling system of two inductive loops (one in antenna module and the second in the microelectronic module) is used to split the device into two independently fabricated components (Figure 24). As mentioned in Section 1.2, the antenna module can be fabricated by embroidering or sewing with conductive threads straight onto a textile material whereas the microelectronic module can be manufactured as an electronic semi-product and then both modules can be integrated together by sewing or embroidering. The possibility of confectioning products with electronic identification tags at the textile factory site and improved resistance to the impact of environmental conditions are the main advantage of the proposed approach to design these RFID devices.

**Figure 25:**
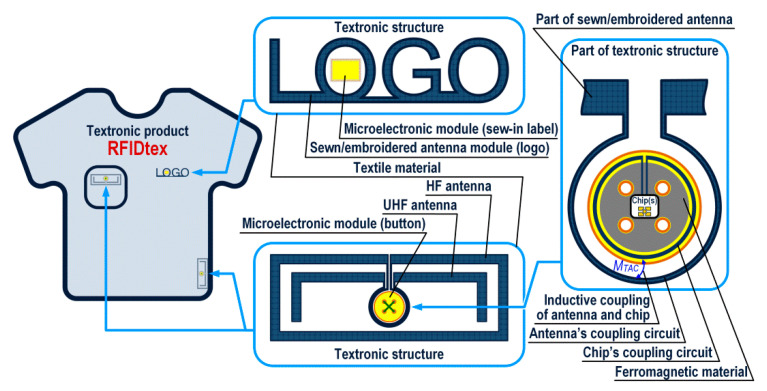
Embodiments of textronic structure with RFID interface (RFIDtex tag).

One of the major prerequisite when designing RFIDtex devices is to develop textile antennas in the way that is invisible to users or the way that meet high aesthetic requirements in order to decorating a consumer good (Figure 25). For example, they can be designed as a logo type of a product producer, aesthetic finish of a pocket flap, decorative seams forming the antenna circuit, etc.

**Figure 26:**
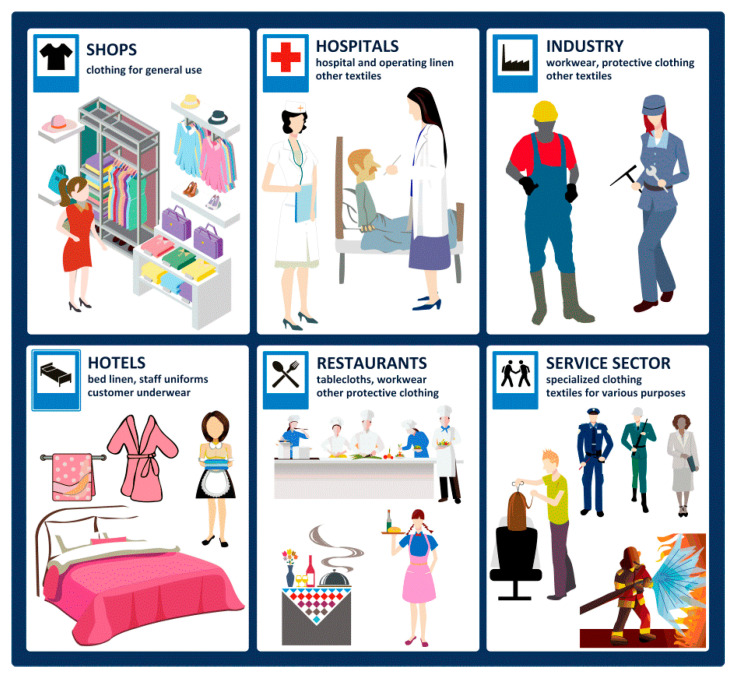
RFIDtex product application areas.

Generally speaking, the RFIDtex product according to the authors’ conception is a typical textile product that is equipped with additional electrical and/or electronic components. The extra components are mainly implemented in order to perform automatic identification function in an RFID system, but they may also include further enhancements such as sensors, indicators, storage of producer or seller data. The RFIDtex conception is dedicated to label textile consumer goods over their whole life cycle (i.e., during production, storage, distribution, operation, maintaining service, and utilization, etc.), where flexibility, comfort of maintaining, esthetic design, and resistance to the impact of environmental conditions (washing, chemical cleaning, or ironing) are welcome. It can be used in all sectors of the economy where the textiles are used: in shops, hospitals, hotels, restaurants, services, etc. (Figure 26).

**Figure 27:**
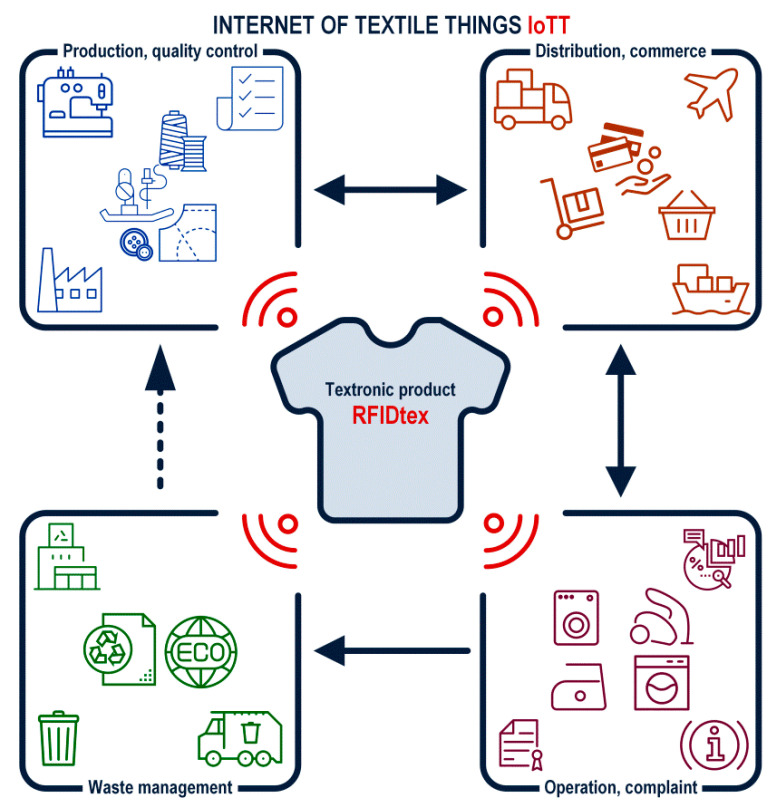
Internet of Textile Things IoTT.

The RFIDtex conception is excellent for supporting the world of Internet of Textile Things (IoTT), because the textronic transponders are intended to be used in the whole life cycle of a labeled object/product (Figure 27).

For example, at the production stage, the quality control may be based on the RFIDtex tags, then the same devices can be implemented in the inventory system of a storage, next they can be used for logistics and adverting tasks in a wholesale and at retail distribution stage, and finally, the stored information about constructional materials can be utilized for safe waste processing. An identification system so conceived in this way can also be implemented in household appliances, for example, for automatic recognition of the type of material during ironing or washing (especially in public washhouse).

## Figures and Tables

**Table 1:** Result of impedance measurements for AMS SL900A chip.

	Passive Mode		Semi-Passive Mode	
*f*_0_ (MHz)	*Z_TC_* (Ω)	*P_Tmin_* (dBm)	*Z_TC_* (Ω)	*P_Tmin_* (dBm)
866	14.9 − j342	−13.1	14.7 − j338	−14.8
915	15.3 − j313	−13.4	14.9 − j309	−14.9

**Table 2:** Parameters of the ISO/IEC 18000-63 communication protocol.

Parameter	Value
Forward link	DSB-ASK, Tari = 25 µs
Return link	FM0, 40 kHz
Command	Query

## Data Availability

All calculated and measured data will be provided upon request to the correspondent authors by email with appropriate justification.
